# TLC-Bioautography and GC-MS Analyses for Detection and Identification of Antioxidant Constituents of *Trachyspermum copticum* Essential Oil

**Published:** 2014

**Authors:** Bahman Nickavar, Abrisham Adeli, Azar Nickavar

**Affiliations:** a *Department of Pharmacognosy, School of Pharmacy, Shahid Beheshti University of Medical Sciences, Tehran, Iran. *; b *Aliasghar Childern’s Hospital, Iran University of Medical Sciences, Tehran, Iran.*

**Keywords:** *Trachyspermum copticum*, Essential oil, Antioxidant activity, Chemical composition

## Abstract

The present work was designed to study the antioxidant activity and to identify the main active components of the essential oil of ajowan (*Trachyspermum copticum*) fruit. GC and GC-MS analyses of the essential oil showed the presence of eight compounds. The main constituents of the oil were thymol (43.7%), *p*-cymene (26.8%), and γ-terpinene (24.9%). The antioxidant and free radical scavenging activities of ajowan oil was evaluated by using ABTS^•+^ and β-carotene bleaching assays. The oil exhibited a considerable dose-dependent antioxidant activity. Antioxidant activity guided fractionation of the oil was carried out by TLC-bioautography method based on the DPPH^•^ assay to screen and separate the main active constituents. The bioautography screening and fractionation resulted in the separation of the main antioxidant compound which was identified as thymol.

## Introduction

Antioxidants are substances that inhibit or delay the oxidation processes. Therefore, they are able to protect the human body, foods, and drugs from oxidative damages (1, 2). Due to the benefits of antioxidants, food, and pharmaceutical products are normally enriched with synthetic antioxidants such as BHA, BHT, *etc*. However, most of the compounds have side effects (3-6). Hence, strong restrictions have been mandated for their application and there is also a trend to the development of more effective and safer antioxidants, especially from natural origins (2, 7). 

Plants have significant antioxidant activities due to the presence of different compounds like polyphenols, flavonoids, terpenoids, *etc *(8-10). For example, various plant essential oils (such as clove, oregano, rosemary, sage, and lavender) have been reported to exhibit strong antioxidant and lipid protection properties (11). Generally, essential oils are widely used as food flavors and preservatives and extend the shelf life of dishes and processed food products (10, 12). 

*Trachyspermum copticum* (L.) Link [Syn. *Carum copticum* (L.) C. B. Clarck, *Trachyspermum ammi* (L.) Sporague, *Ammi copticum* L.] is an aromatic plant belonging to the Apiaceae family (13). Its fruit (ajowan) has been widely consumed as a food flavoring agent and spice. Besides, the fruits have several therapeutic effects and they have been used for the treatment of colic, flatulence, indigestion, dyspepsia, and diarrhea (14, 15). Some studies have been carried out on the chemical composition of the essential oil from ajowan fruit. The studies indicated that the major compounds of the oil were thymol, γ-terpinene, and *p*-cymene (16-22). Though the content of the volatile oil from *T. copticum* fruit has been investigated, little is known about the antioxidant activity of the oil (23). However, to the best of our knowledge, there is no-study on isolation and identification of active constituents from ajowan oil having antioxidant activity. 

The main objectives of the present study were to evaluate the antioxidant properties of the essential oil from *T. copticum* fruit and to find out which compounds contribute to the effects.

## Experimental

*Plant material*

Ajowan fruit was bought from a local market in Tehran, Iran in summer 2011 and authenticated by M. Kamalinejad at the Herbarium of the Department of Pharmacognosy, School of Pharmacy, Shahid Beheshti University of Medical Sciences where the voucher specimens have been preserved.

*Chemicals*

All of the chemicals used in this study were purchased from Sigma-Aldrich Chemical Co. (France) and/or Merck Company (Germany).

*Essential oil isolation*

The fruit was crushed and subjected to the hydrodistillation for 3 h using a Clevenger type apparatus. The oil was dried over anhydrous sodium sulfate and stored under N_2_ in a dark sealed vial at 4 °C until required.

*Gas chromatography analyses*

GC-FID analyses were carried out on an Agilent GC 7890A gas chromatograph equipped with a FID and a HP-5 capillary column (30 m × 0.25 mm, 0.25 µm film thicknesses). The initial oven temperature was held at 50 °C for 3 min, increased up to 120 °C with a heating rate of 3 °C/min; then the column temperature was programmed as 120 °C to 250 °C by a heating rate of 5 °C/min and held at this temperature for 5 min. The carrier gas was N_2_ with a flow rate of 2 mL/min. The injector temperature and detector temperature were adjusted to 280 °C and 300 °C, respectively. Sample size was 1.0 µL with a split ratio of 1:10.

GC-MS analyses were performed on an Agilent 7890A GC interfaced to an Agilent 7000 triple quad mass spectrometer. The operating conditions were the same conditions as described for GC-FID analyses, but the carrier gas was He. EI-MS spectra were recorded at 70 eV ionization voltage and the mass range was from *m/z* 50 to 1000 amu.

The identification of compounds was accomplished by comparing their mass spectra to those of the Wiley 275.L library as well as their retention indices with those reported in the literature. Retention indices were calculated using the retention times of *n*-alkanes (C_8_ – C_18_). 

*Antioxidant assays*

*ABTS* ^•+^ *assay*

The antioxidant capacity of ajowan volatile oil was evaluated by a method based on the decolonization of radical cation of ABTS^•+^ [2,2’-azinobis-(3-ethylbenzothiazoline-6-sulfonic acid] (24). The ABTS^•+^ radical cation was prepared by the reaction between 7 mM ABTS and 2.45 mM potassium persulfate, after incubation at room temperature for 12-16 h. Prior the assay, the ABTS^•+^ solution was diluted with methanol to get an absorbance of 0.70 ± 0.02 at 734 nm. 4 mL of the diluted ABTS^•+^ solution was mixed with 200 µL of different dilutions of each sample, including positive controls (vitamin C and gallic acid), ajowan oil, and its active compound (thymol). The reaction mixture was allowed to stand at room temperature for 10 min then, the absorbance was recorded at 734 nm. The inhibition of ABTS^•+^ (*I*_ABTS•+_ %) in percent was calculated by the following formula:

$I_{{\mathrm{ABTS}}^{∙+}}\left(\%\right)=100\times [\frac{A_{\mathrm{control}}-(A_{\mathrm{sample}}-A_{\mathrm{blank}})}{A_{\mathrm{control}}}]$

where *A*_sample_*, A*_blank_*,* and *A*_control_ were the absorbance of sample, blank, and control, respectively.

*Linoleic acid/β-carotene bleaching assay*

The antilipid peroxidation activity of ajowan essential oil was determined by the linoleic acid/β-carotene model (24). A mixture of β-carotene and linoleic acid was prepared with 2 mL of a 200 µg/mL solution of β-carotene in chloroform, 45 µL of linoleic acid and 400 mg of Tween 40. Chloroform was evaporated under vacuum then, 100 mL of oxygenated distilled water was added to the residue. 0.5 mL of various dilutions of each sample, including positive controls (vitamin C and gallic acid), ajowan oil, and its active compound (thymol), was added to 4.5 mL of the above mixture and the emulsion system was incubated in a hot water bath at 50 °C for 2 h. The initial absorbance at 470 nm (t = 0) for each reaction mixture was measured immediately. Subsequent absorbance values were obtained after incubation. The inhibition percentage of bleaching (*I*_bleaching_ %) was calculated using the following equation:

*I*_bleaching_ (%) = (Absorbance of sample after 2 h of assay / Initial absorbance of sample) × 100

*Rapid screening for antioxidants*

For screening of antioxidant compounds in ajowan essential oil, the TLC-bioautography method was carried out (9, 25). The diluted oil (1:20 in methanol) was spotted on silica gel sheets (silica gel 60 F_254_ TLC plates) and developed in *n*-hexane-ethyl acetate (9:1). Plates were sprayed with the methanolic solution of DPPH^•^ (0.2%). The active constituents were detected as yellow spots on a violet background. Only zones where their color turned from violet to yellow within the first 30 min (after spraying) were taken as positive results. 

*Activity guided fractionation of the essential oil for antioxidants*

For the isolation and identification of the active compounds in the essential oil, PTLC was performed using the conditions previously described (9). The regions showing DPPH^•^ scavenging activity were scrapped off then, they were eluted with chloroform. All resulting constituents were analyzed by GC-FID and GC-MS and also tested for their antioxidant activities.

*Statistical analysis*

All the experiments were carried out in triplicate. IC_50_ values [inhibitory concentration (µg/mL)] were calculated from logarimic regression curves (*I *% against sample concentration) and presented with their respective 95% confidence limits. The one-way ANOVA followed by Tukey’s post test was used for comparisons. A probability value of *p* < 0.001 was considered to denote a statistically significant difference. All the statistical analyses were accomplished using the computer software GraphPad Prism 3.02 for Windows (GraphPad Software, San Diego, CA, USA).

## Results and Discussion

**Figure 1:**
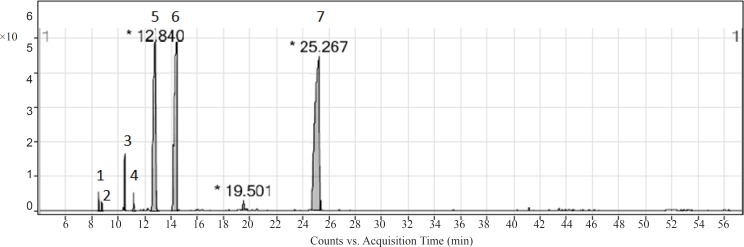
GC-MS chromatogram of ajowan essential oil. The identified compounds have been listed in Table 1

The hydrodistillation of ajowan fruit gave yellowish oil with a yield of 2.5% (± 0.1%) v/w. Eight compounds (representing of 99.3% of the total constituents) were identified in the oil by GC analyses (Figure 1). The identified compounds and their percentages have been given in Table 1. The oil consisted mainly of hydrocarbon monoterpens (55.3%) and oxygenated monoterpenoids (44.0%). The major compounds were thymol (43.7%), *p*-cymene (26.8%), and γ-terpinene (24.9%). The composition of the oil was similar to those reported in literature (16-22). 

**Table 1:** Chemical composition of ajowan essential oil

**No.**	**Compound**	**RRI**	**Percentage**
1	α-Thujene	927	0.6
2	α-Pinene	934	0.3
3	β-Pinene	976	2.0
4	β-Myrcene	990	0.7
5	*p*-Cymene	1029	26.8
6	γ-Terpinene	1064	24.9
7	Thymol	1299	43.7
8	Carvacrol	1302	0.3

The antioxidant capacity of the essential oil from ajowan fruit was quantified with two different assays, including ABTS^•+^ and β-carotene/linoleic acid methods. Generally, the use of multiple measurements provides a better insight into the antioxidant potential of natural products. On the other hand, these two techniques are rapid and reliable methods to study the free radical scavenging and antioxidant activities of plant products (1, 26). 

**Figure 2:**
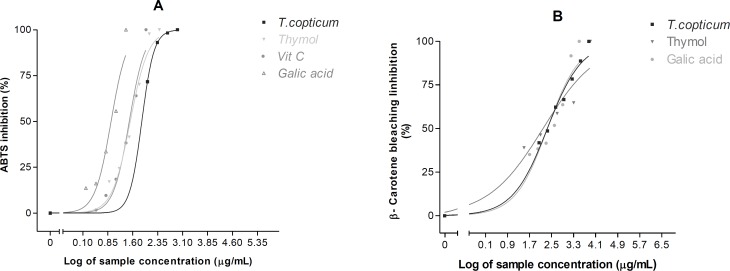
Concentration-dependent free radical scavenging and antioxidant activities of ajowan essential oil and its active constituent determined using (A) ABTS^•+^ method and (B) Linoleic acid/β-carotene bleaching technique. Each point represents the mean of three experiments and vertical bars represent the SEM

The ABTS^•+^ decolonization test is widely employed to assess the antioxidant activity of natural sources. In this method, antioxidant molecules can scavenge ABTS^•+^ radical cations by providing hydrogen atom or electron donation (27). Ajowan essential oil demonstrated a sigmoidal dose-response curve over the concentration range tested in the ABTS^•+^ assay (Figure 2A). The IC_50_ value was 74.78 (64.58 – 86.60) μg/mL (Table 2). 

The β-carotene bleaching method is a commonly test for determining of antioxidants to inhibit the lipid peroxidantion in the propagation phase (28). Ajowan oil showed a lipid peroxidation inhibitory activity in a concentration-dependent manner (Figure 2B). The IC_50_ value was found to be 221.90 (185.30 – 265.80) μg/mL (Table 2). 

**Table 2:** Free radical scavenging activity and antioxidant potency of ajowan essential oil and its active constituent

**Sample**	**IC**_50 (ABTS•+)_ (µg/mL)*	**IC**_50 (β-carotene bleaching)_ (µg/mL)*
Ajowan essential oil	74.78 (64.58 – 86.60)^c^	221.90 (185.30 – 265.80)^a^
Thymol	33.57 (29.53 – 38.17)^b^	154.90 (87.04 – 275.60)^a^
Gallic acid	8.90 (7.55 – 10.50)^b^	222.80 (168.60 – 294.60)^a^
Vitamin C	31.38 (28.24 – 34.87)^a^	-

^(^^a-c^^)^ Different superscript letters within each column denote significant differences (Tukey’s test, *p* < 0.001).

* IC_50_ values have been presented with their respective 95% confidence limits.

These findings are in accordance with those of Kavoosi *et al*. (2013) who reported that *Carum copticum* essential oil had noticeable free radical scavenging and antioxidant activities (23).

**Figure 3:**
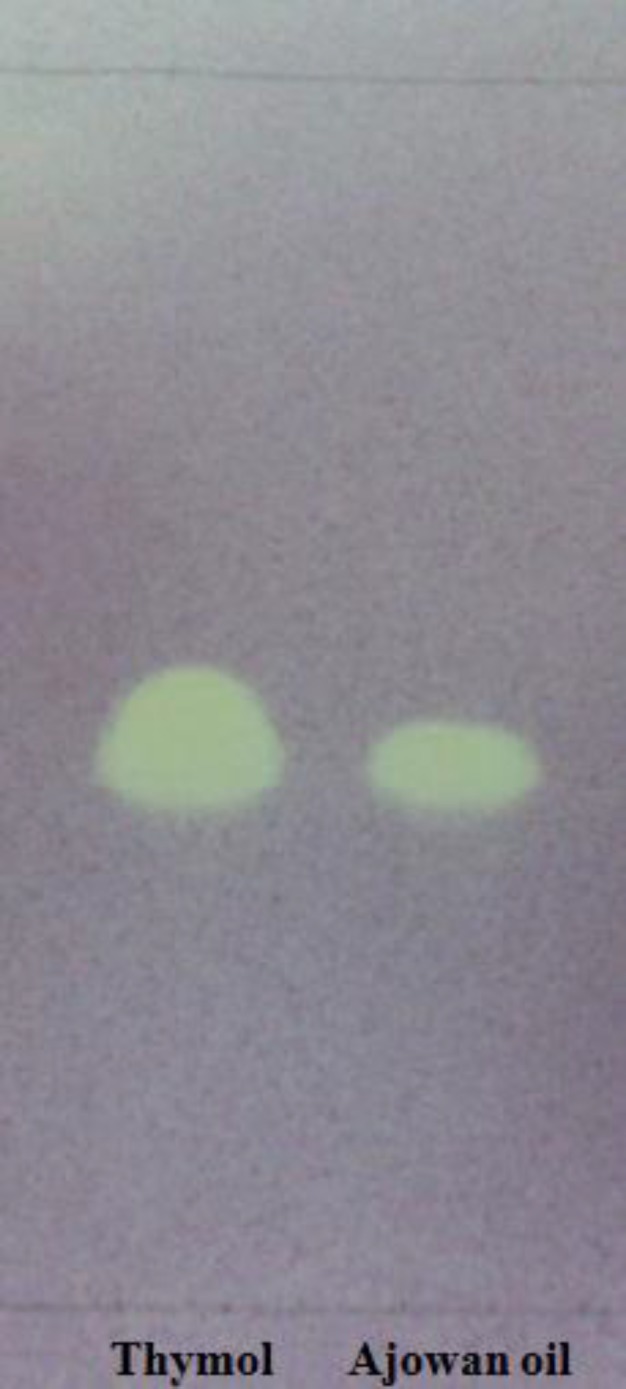
TLC plate stained with 0.2% DPPH^•^ solution

Because of high antioxidant and free radical scavenging activities of ajowan essential oil, it was further investigated to identify its active constituents. Therefore, a preliminary screening was initially carried out using the dot-blot DPPH^•^ staining method on TLC. The application of ajowan oil in the bioautography system mentioned above showed one active band (F_1_) with R_f_ value of 0.44 (Figure 3). 

**Figure 4:**
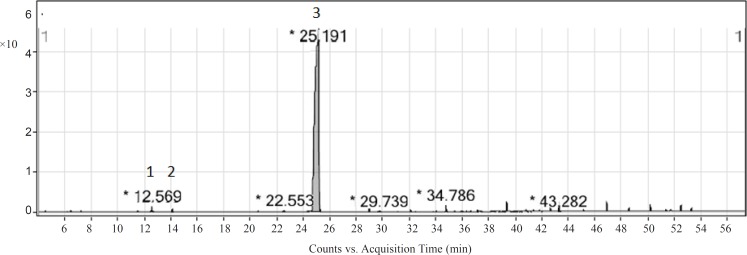
GC-MS chromatogram of F_1_ active band obtained from ajowan essential oil. The identified compounds have been listed in Table 3

As the essential oil presented a significant antioxidant activity in the assays and bioautography test, it was subjected to the PTLC for isolation of the active compounds. Hence, F_1_ band was scratched out and eluted with chloroform and the compound(s) present in it was identified by GC-MS. Four compounds were identified in F_1_ band (Figure 4). Components identified in this band with their relative percentages have been showed in Table 3. The major compound found in the active band was thymol (98.6%). The compound exhibited a significant radical scavenging activity [IC_50 (ABTS•+)_ = 33.57 (29.53 – 38.17) μg/mL] and antilipid peroxidation [IC_50 (β-carotene bleaching)_ = 154.90 (87.04 – 275.60) μg/mL]. Other studies also showed that thymol had a high antioxidant activity (26, 29, 30). 

**Table 3:** Chemical composition of F_1_ active band obtained from ajowan essential oil

**No.**	**Compound**	**Percentage**
1	*p*-Cymene	trace
2	γ-Terpinene	trace
3	Thymol	98.6
4	Carvacrol	trace

## Conclusion

The overall, ajowan fruit essential oil studied here exhibited potent free radical scavenging and antioxidant activities in a series of *in-vitro* tests. The bioautography screening and separation of antioxidant compounds led to the identification of thymol as the major antioxidant constituent of the oil.
